# Targeting Cancer Stem Cells through Epigenetic Modulation of Interferon Response

**DOI:** 10.3390/jpm12040556

**Published:** 2022-04-01

**Authors:** Jau-Ling Huang, Si-Yun Chen, Chang-Shen Lin

**Affiliations:** 1 Department of Bioscience Technology, College of Health Science, Chang Jung Christian University, Tainan 711, Taiwan; jaulingh@mail.cjcu.edu.tw; 2 Graduate Institute of Medicine, College of Medicine, Kaohsiung Medical University, Kaohsiung 807, Taiwan; qoxoruby@gmail.com; 3 Center for Cancer Research, Kaohsiung Medical University, Kaohsiung 807, Taiwan; 4 Department of Medical Research, Kaohsiung Medical University Hospital, Kaohsiung Medical University, Kaohsiung 807, Taiwan; 5 Department of Biological Sciences, National Sun Yat-sen University, Kaohsiung 804, Taiwan

**Keywords:** cancer stem cells, cytosolic nuclei acids, epigenetic and immune checkpoint inhibitors, interferon response

## Abstract

Cancer stem cells (CSCs) are a small subset of cancer cells and are thought to play a critical role in the initiation and maintenance of tumor mass. CSCs exhibit similar hallmarks to normal stem cells, such as self-renewal, differentiation, and homeostasis. In addition, CSCs are equipped with several features so as to evade anticancer mechanisms. Therefore, it is hard to eliminate CSCs by conventional anticancer therapeutics that are effective at clearing bulk cancer cells. Interferons are innate cytokines and are the key players in immune surveillance to respond to invaded pathogens. Interferons are also crucial for adaptive immunity for the killing of specific aliens including cancer cells. However, CSCs usually evolve to escape from interferon-mediated immune surveillance and to shape the niche as a “cold” tumor microenvironment (TME). These CSC characteristics are related to their unique epigenetic regulations that are different from those of normal and bulk cancer cells. In this review, we introduce the roles of epigenetic modifiers, focusing on LSD1, BMI1, G9a, and SETDB1, in contributing to CSC characteristics and discussing the interplay between CSCs and interferon response. We also discuss the emerging strategy for eradicating CSCs by targeting these epigenetic modifiers, which can elevate cytosolic nuclei acids, trigger interferon response, and reshape a “hot” TME for improving cancer immunotherapy. The key epigenetic and immune genes involved in this crosstalk can be used as biomarkers for precision oncology.

## 1. Introduction

**Figure 1:**
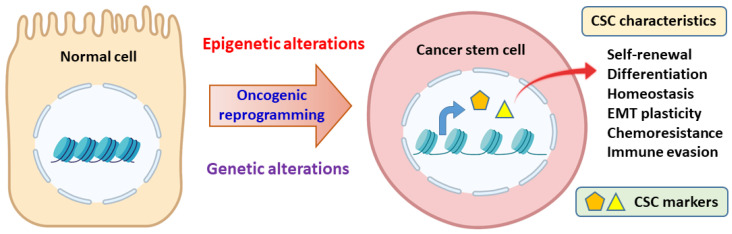
The characteristics of cancer stem cells (CSCs). CSCs can emerge through acquiring genetic and epigenetic alterations that leads to aberrant expressions of CSC markers and allows CSCs to evade immunosurveillance and resist anticancer therapy. EMT, epithelial-mesenchymal transition. Graph created with Biorender.com (accessed on 2 March 2022).

Cancer stem cells (CSCs) are a small subset of cancer-initiating and/or cancer maintenance cells in the cancer mass. CSCs exhibit similar characteristics as that of normal stem cells, such as self-renewal, differentiation, and homeostasis. However, CSCs usually acquire genetic and epigenetic alterations that allow CSCs to evade tumor suppression mechanisms (Figure 1) [1,2,3,4]. For example, CSCs can enter into dormancy to escape from immunosurveillance and anticancer therapy [5]. Therefore, many conventional anticancer therapeutics fail to completely eradicate CSCs, leading to cancer relapse and metastasis [6,7,8]. To overcome this as of yet unresolved clinical need, a better understanding of CSC characteristics is essential for the design of CSC eradication strategy.

Immune evasion is a fundamental feature of CSCs, which is a significant obstacle in immune checkpoint blockade (ICB) therapy [9,10,11]. CSCs can downregulate expression of tumor-associated antigens and suppress antigen presentation by the major histocompatibility complex class I (MHC-I) to cytotoxic CD8+ T cells [12]. CSCs can also suppress the activity of natural killer (NK) cells, which play an important role in antitumor immunosurveillance [13]. Besides, CSCs may upregulate the immune checkpoint ligands, such as programmed death-ligand 1 (PD-L1), and thereby decrease the efficacy of immune checkpoint inhibitors (ICIs) and inhibit cytotoxic activity of CD8+ T cells [14]. These CSC-mediated immune evasion mechanisms sculpt the immunosuppressive or “cold” tumor microenvironment (TME), marked by the decreased infiltration of functional CD8+ T cells [15]. Therefore, CSC should be considered a relevant therapeutic target in immune-oncology.

Unlike bulk tumor cells, CSCs exhibit distinct epigenetic landscapes that are marked by altered DNA methylation and histone modifications [16,17]. These epigenetic changes are characterized by numerous epigenetic modifiers, such as methyltransferases/demethylases, acetyltransferases/deacetylases, and a lot of reader proteins and non-coding RNAs [18]. Alterations of epigenetic modifiers may contribute to CSC-mediated immune evasion [19]. Therefore, these epigenetic modifiers are candidate CSC biomarkers and therapeutic targets [20]. Indeed, recent studies have shown that the inhibition of certain epigenetic modifiers, such as the lysine-specific demethylase 1 (LSD1/KDM1A), can suppress CSCs and improve ICB therapy [21,22,23]. Notably, these epigenetic modifiers are essential to silencing endogenous retrovirus (ERV) elements in somatic and stem cells including CSCs [24,25,26,27]. The inhibition of these epigenetic modifiers results in the de-repression of ERV elements and induction of interferon response [28,29]. Interferons play a central role in regulating innate and adaptive immune responses and thus influence the interaction between CSCs and immune TME, which determines the outcome of ICB therapy [30,31,32]. In this review, we introduce the interplay between CSCs and interferon response, provide some examples to demonstrate the roles of epigenetic modifiers in contributing to CSCs characteristics, and discuss the inhibition of epigenetic modifiers as a potential strategy to induce interferon response for improving ICB therapy. It is clear that these epigenetic modifiers can be biomarkers for CSCs.

## 2. The Interferon Response and Immune Checkpoint Blockade Therapy

### 2.1. The Interferon Signaling

**Figure 2:**
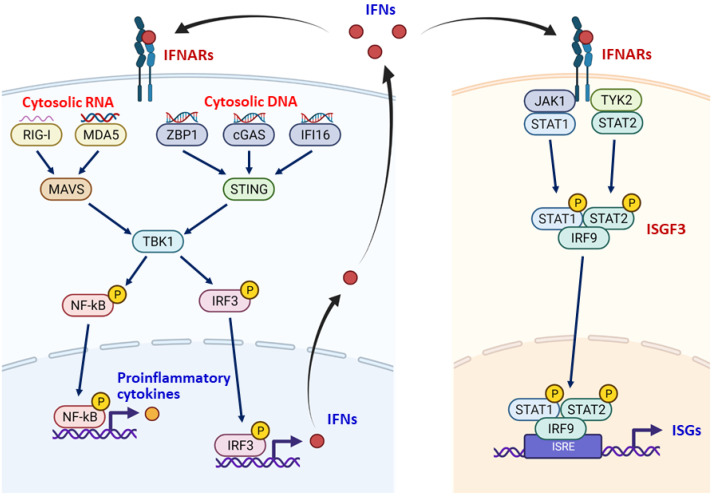
The interferon response. The type I interferons (IFNs, red dots) are induced by the detection of cytosolic DNA and RNA by various pattern-recognition receptors (ZBP1, cGAS, IFI16, RIG-I, MDA5), which then activate TBK1 through STING and MAVS (left part). The TBK1 phosphorylates IRF3 and NF-kB to activate their transactivation activities toward the promoters of IFNs and pro-inflammatory cytokine genes. The induced type I interferons are secreted outside of the cells to bind to their receptors (IFNARs) on the cell membranes of self and neighbor cells. The binding of IFNs to IFNARs activates JAK1 and TYK2 to phosphorylate STAT1 and STAT2, facilitating the formation of interferon-stimulated gene factor 3 (ISGF3) complex and subsequently transactivation of type II interferon and numerous interferon-stimulated genes (ISGs). The expressed IFNs, ISGs, and pro-inflammatory cytokines are critical for the induction of innate and adaptive immune responses. ISRE, interferon-stimulated response element. Graph created with Biorender.com.

The type I interferons (IFNs), including IFN-α and IFN-β, are innate cytokines that are equipped to every cell as the first-line immune response to combat invaded pathogens. Upon infection, the pathogenic nucleic acids can be detected by a variety of pattern recognition receptors (PRRs), which then transmit these “danger signals” through STING-TBK1 and MAVS-TBK1 axes to activate the hub transcription factors NF-kB and interferon regulatory factor 3 (IRF3), thereby allowing these transcription factors to translocate into the nucleus and turn on the gene expressions of type I interferons and pro-inflammatory cytokines. Depending on the nature of nucleic acids in the cytosol, the cells use different PRRs to detect viral DNA, such as cGAS, IFI16, DDX41, AIM2, ZBP1/DAI, RNA polymerase III, and viral RNA, such as DDX58/RIG-I, IFH1/MDA5, and Toll-like receptors (TLRs) 3, 7, 8, 9 [33] (Figure 2). In addition to viral nucleic acids, self-cytosolic nucleic acids can also trigger interferon response. The sources of self-cytosolic nucleic acids include DNA damage fragments from the nucleus or mitochondria, leaky DNA from micronucleus, and viral mimicry RNA from ERV elements [28,34,35,36,37].

The induced type I interferons, which are secreted from virus infected or malignant cells, stimulate the expression of type II interferon (IFN-γ) and numerous interferon-stimulated genes (ISGs) by binding to the type I interferon receptors (IFNAR1/IFNAR2) on the cell membrane. The interferon-bound IFNAR1/IFNAR2 activate the Janus kinase 1 (JAK1) and Tyrosine kinase 2 (TYK2), which phosphorylate the interferon-stimulated gene factor 3 (ISGF3) composed of interferon regulatory factor 9 (IRF9), signal transducer and activator of transcription 1 (STAT1), and STAT2 (Figure 2). Because almost all cells, especially immune cells, express IFNAR1/IFNAR2, type I interferons play a central role in the regulation of innate and adaptive immune cells to deal with infections as well as malignancies [31,38].

The dendritic cells (DCs) and natural killer (NK) cells are well-documented innate immune cells that produce pro-inflammatory cytokines and chemokines in response to stimulation by type I interferons. These pro-inflammatory cytokines, such as IFN-γ and tumor necrosis factor (TNF), and chemokines, such as CXCL10, are critical for modulating adaptive immune response to inhibit cancer cells in the TME. For example, CXCL10 can attract the cytotoxic CD8+ T cells to the TME for cancer killing [39], and the high expression of CXCL10 in the TME can predict a good prognosis in oral cancer patients [40]. In addition to DCs and NK cells, type I interferons can also regulate various immune cells, such as macrophage, B cells, T cells, and stromal cells in the TME [31,41].

### 2.2. The Immune Checkpoint and Cancer Immunotherapy

The cytotoxic CD8+ T cells are the major effector cells that kill cancer cells in cancer immunotherapy. The activation of cytotoxic CD8+ T cells requires specific antigens that are processed and presented together with MHC-I on the cell surface of professional antigen-presenting cells (APCs), such as DCs. In addition to MHC-I, co-stimulatory signals provided by the B7 molecules of APCs are also required. The B7 molecules bind to the CD28 of CD8+ T cells and induce T-cell activation and proliferation. In contrast, T-cell activity is suppressed by inhibitory checkpoint molecules, such as PD-1 and cytotoxic T-lymphocyte antigen-4 (CTLA-4), to avoid over-activation, which may lead to tissue damage and autoimmune diseases. Cancer cells can express the ligands of T-cell inhibitory molecules, such as PD-L1 and PD-L2, to inhibit the activation of CD8+ T cells and escape from immune attack. In this way, antibodies (ICIs) that block the actions of PD-1, PD-L1, and CTLA-4 are developed to activate CD8+ T cells in the TME for cancer immunotherapy. However, the efficacies of ICIs are limit in many cancers due to the “cold” TME, where is an immunosuppressive territory and is short of activated CD8+ T cells and pro-inflammatory cytokines/chemokines [15]. Therefore, additional managements that can reverse the “cold” TME to the “hot” one are essential to improve ICB therapy. For example, various strategies have been applied to induce cytosolic nucleic acids in cancer cells, which can trigger an interferon response and sculpt a “hot” TME to improve ICB therapy [35,36,42].

## 3. The Interplay between Interferon Response and CSCs

The interferon response is critical for antitumor immunosurveillance. In addition, interferons and ISGs have been demonstrated to inhibit CSC characteristics, such as chemoresistance. However, CSCs may evolve various mechanisms to evade interferon-mediated immunosurveillance [32]. The interplay between interferon response and CSCs are introduced below.

### 3.1. The Inhibitory Effects of Interferon Response on CSC Features

Doherty et al. have reported that the mesenchymal breast CSCs exhibit a significantly repressed IFN/STAT gene expression signature [43]. The treatment of these breast CSCs by IFN-β induces a less aggressive epithelial/non–CSC state, which is evident by the re-expression of the epithelial/non–CSC marker (CD24) and downregulation of mesenchymal markers (VIMENTIN, SLUG), concomitant with reduced abilities of cell migration and tumor-sphere formation. According to this IFN-β-induced gene signature, Doherty et al. demonstrate that the triple-negative breast cancer (TNBC) patients with this IFN-β metagene signature have an improved survival rate, accompanied by increased tumor-infiltrating lymphocytes and a repressed CSC metagene signature in their tumor tissues. Thus, IFN-β can induce epithelial differentiation program and repress CSC properties in TNBC [44].

In the 4T1 mouse model of breast cancer, the amounts of IFN-γ and CD8+ T cells decrease but the levels of ALDH+ breast CSCs increase along with tumor growth. The administration of IFN-γ can suppress the sphere formation of 4T1 tumor in vitro and decrease the number of ALDH+ breast CSCs in the 4T1 tumor-bearing mice [45]. Moreover, IFN-α can potentiate the anti-proliferative and apoptotic effects of epigenetic drugs in CSCs of colorectal cancer [46]. The inhibition of glioblastoma CSCs by IFN-α is also reported [47].

Both IFN-β and IFN-γ can induce cell cycle arrest and the dormancy of tumor-repopulating cells (TRCs) or disseminated tumor cells (DTCs), which exhibit CSC characteristics and can re-initiate tumor growth at distant organs long after therapy. For example, Liu et al. reported that IFN-β or IFN-γ treatment induces melanoma TRCs to enter dormancy via an indolamine 2,3-dioxygenase 1-kynurenine-aryl hydrocarbon receptor-p27-dependent pathway. Moreover, the high expression of IFN-β is correlated with a longer survival in melanoma patients [48,49]. Lan et al. also demonstrated that chemotherapy elicits an interferon response and induces the dormancy of breast cancer cells. A loss of IFN-β production in cancer cells leads to an escape from dormancy. Importantly, the breast cancer patients with high levels of circulating IFN-β exhibit a longer distant metastasis-free survival [50]. Using single-cell transcriptomics and ex vivo profiling, Owen el al. have shown that tumor-intrinsic type I interferon response is activated in dormant bone metastases but is suppressed in proliferating prostate cancer cells in the bone. In the prostate cancer patients with bone metastasis, the tumor-intrinsic type I interferon response is suppressed when compared to that in primary tumors [51]. By examining the liver metastasis of breast cancer cells, Correia et al. identified IFN-γ as the key factor to control the dormancy of disseminated tumor cells (DTCs). The proportion of IFN-γ-positive NK cells is higher in the dormant stroma than that in normal liver tissues. In contrast, the percentage of IFN-γ-expressing NK cells decreased in the metastatic sites when compared to that in normal tissues. Moreover, the in vitro treatment of IFN-γ can increase the fraction of quiescent DTCs [52]. These results support that IFN-β and IFN-γ can inhibit the outgrowth of DTCs and TRCs by promoting these cells to enter a dormancy state.

In addition to interferons, many interferon-related genes can inhibit CSCs. The interferon-stimulated gene 15 (ISG15) is an ubiquitin-like protein, which plays pleiotropic roles in the TME. Furthermore, ISG15 can inhibit the protein translation of ABCC2 and increase drug sensitivity in cisplatin-resistant ovarian cancer cells [53]. Moreover, ISG15 inhibits CSC-like phenotypes of cisplatin-resistant ovarian cancer cells and suppresses tumor formation in nude mice. In contrast, the inhibition of ISG15 expression in cisplatin-sensitive ovarian cancer cells induces CSC-like features. In ovarian cancer patients, low ISG15 expression predicts poor prognosis [54]. However, Chen et al. reported a contradictory role of ISG15 in nasopharyngeal carcinoma (NPC), in which high ISG15 expression promotes the CSC phenotype and predicts a poor prognosis in NPC patients [55]. Therefore, the effects of ISG15 on CSCs may be tumor context-dependent.

Another drug resistant gene, ABCG2, can be repressed by the interferon regulatory factor 6 (IRF6) [56]. As is known, IRF6 directly binds to the promoter of ABCG2 and suppresses its expression in NPC cells. As a result, IRF6 enhances cell sensitivity to chemotherapeutic drugs and inhibits CSC properties of NPC cells. In clinical NPC specimens, downregulation of IRF6 is found to be correlated with elevated ABCG2 levels [56]. Furthermore, decreased IRF6 expression due to DNA hypermethylation is observed in kidney cancer, which predicts poor prognosis in the patients [57].

Huang et al. reported that the interferon-induced protein 44-like (IFI44L) can decrease the chemoresistance of hepatic CSCs towards doxorubicin. In contrast, depletion of IFI44L promotes cell migration, invasion, and pulmonary metastasis of hepatic CSCs. Downregulation of IFI44L is frequently observed in hepatocellular carcinoma (HCC) and is correlated with patient’s poor survival [58]. These results suggest that the interferon response plays a crucial role in the suppression of CSCs.

However, the interferon response may potentiate CSC properties in pancreatic adenocarcinoma and sarcoma [59,60,61]. Therefore, interferon response presents a double-edged sword, which either inhibits or promotes CSCs depending on the TME and tumor contexts [30,32,62]. For example, a low level of IFN-γ stimulates CSC properties in non-small cell lung cancer (NSCLC) cells; however, a high concentration of IFN-γ induces apoptosis through the JAK1/STAT1/caspase pathway [63]. Thus, precise clarification of interferon response in different settings is important for the development of a CSC eradication strategy.

### 3.2. Evasion of Interferon Response by CSCs

Several studies have shown that CSCs are able to evade interferon-mediated tumor suppression [9,10]. In addition to the characteristics of self-renewal and epithelial-mesenchymal transition (EMT), it has been shown that breast CSCs exhibit a significantly repressed interferon gene expression signature, suggesting that interferon response is impaired in breast CSCs [43]. Indeed, in a HER2/Neu transgenic mouse model of breast cancer, a disruption of type I interferon signaling results in early onset of breast cancer, which exhibits an increased number of breast CSCs with an enhanced clonogenic activity and expression of stemness markers [64].

The precise mechanisms underlying the inhibition of the interferon response in CSCs remain to be elucidated; however, some CSC-related markers may be involved in this suppression. Li et al. have reported that CD133-enriched HCC cells are resistant to IFN-γ-induced autophagy and growth suppression [65]. Oncostatin-M and osteopontin both stimulate CSC properties, can inhibit interferon signaling in breast and hepatic CSCs [44,66]. Another CSC-promoting factor ETV7, which is a member of ETS transcription factor family, can enhance CD44+/CD24^low^ breast CSCs properties, such as tumor sphere formation, cell plasticity, and resistance to chemotherapy and radiotherapy. Notably, an interferon-responsive gene signature is downregulated in ETV7-expressing breast cancer cells. In breast cancer patients, the low expressions of ETV7-repressed interferon signature genes are associated with worse prognoses [67], suggesting that CSC-mediated suppression of interferon response is correlated with patient’s poor outcomes.

Glioma CSCs is able to evade interferon response via the downregulation of STAT1, a critical transcription factor for the induction of ISGs (Figure 2). Mechanistically, the epigenetic regulator MBD3, which is preferentially expressed in glioma CSCs, recruits the NuRD repressive complex to the STAT1 promoter and inhibits its expression by histone deacetylation. As a result, glioma CSCs escape from interferon-mediated immunosurveillance [68].

The Moloney murine leukemia virus insertion site 1 (BMI1) is a CSC marker found in several cancers [69]. Furthermore, BMI1 binds directly to the promotor region of interferon regulatory factor 7 (IRF7) and represses its expression, leading to a suppression of the interferon response. The inhibition of IRF7-regulated interferon signaling by BMI1 can be enhanced by the interaction between BMI1 and BTF3 (Basic transcription factor 3), which is a component of the RNA polymerase II transcription complex. Overexpression of BTF3 stabilizes BMI1 and promotes stemness, EMT, cell migration and proliferation in prostate, colorectal, and TNBC cells [70,71,72]. In contrast, knockdown of BTF3 activates interferon signaling in cancer cells [70].

The miR-199a is enriched in mammary stem cells (MaSCs) and in breast CSCs. In addition to stimulating CSC features, miR-199a represses the interferon response by directly targeting the nuclear receptor corepressor LCOR, which is able to sensitize MaSCs and CSCs to interferon-induced differentiation and senescence. A high expression of miR-199a is associated with poor relapse-free survival in patients with breast cancer, especially in those with estrogen receptor-negative breast cancers, which exhibit enhanced CSC properties [73]. These studies demonstrate that several CSC-related markers can evade interferon-mediated anticancer effects via multiple mechanisms.

## 4. Epigenetic Modifiers Contribute to CSCs

The interferon response can be induced by the de-repression of ERV elements or DNA damage-induced cytosolic nucleic acids [28,34,35,36,37], and both events can be regulated by specific methyltransferases and demethylases. Here, we focus on the roles of histone methylation modifiers LSD1, BMI1, G9a, and SETDB1 in CSC characteristics, and discuss the recent advances in the targeting of these epigenetic modifiers in the next section, as a novel strategy to induce interferon response for the improvement of ICB therapy.

### 4.1. LSD1

Interestingly, LSD1 removes mono- and di-methyl groups (me1/2) from lysine 4 (K4) and lysine 9 (K9) of histone H3, thus alters chromatin configuration and gene expression [74]. In addition to histones, LSD1 can demethylate non-histone proteins, such as OCT4 and DNMT1, and regulate global DNA methylation and cell stemness [75,76]. Furthermore, LSD1 is essential to keep stemness in various cancers and mediate chemoresistance in breast and liver cancers [22,77,78,79]. In addition, LSD1 activation promotes EMT and modulates the TME in breast cancer [80]. LSD1 inhibits the activation of ERV elements through demethylation of the Argonaute RISC catalytic component 2 (AGO2), leading to diminished ERV-expressed cytosolic RNA and repressed interferon-mediated antitumor immunity [21]. Besides, LSD1 can inhibit the functions of p53 and RB to stimulate cell proliferation [81,82]. The overexpression of LSD1 is correlated with poor patient outcome in several cancers, including leukemia, prostate, lung, brain, and breast cancers [78,83,84]. Recently, Zhao et al. found that LSD1 delivered via small extracellular vesicles promotes gastric cancer cell stemness [85].

### 4.2. BMI1

With regard to BMI1, it is an essential component of the polycomb repressive complex 1 (PRC1) that inhibits gene expression by modifying histone H2A with ubiquitin [86]. It plays an important role in the self-renewal of hematopoietic and neural stem cells [87,88]. A high expression of BMI1 contribute to CSC characteristics, such as self-renewal, EMT, metastasis, and chemoresistance [69]. In head and neck squamous cell carcinoma (HNSCC), BMI1 is highly expressed in CD44+ cells that exhibit CSC features of self-renewal, differentiation, and chemoresistance [89,90]. BMI1 also preferentially expresses in the side population (SP) of HCC cells and contributes to the maintenance of tumor-initiating ability of SP cells in an immunodeficient mouse mode [91]. Cui et al. have found that BMI1 highly expresses in neuroblastoma and contributes to the tumorigenicity of neuroblastoma cells by suppressing apoptosis [92]. Furthermore, BMI1 can regulate tumor-initiating capacity of CD133+ glioblastoma stem cells via the activation of integrin alpha 2-associated gene networks [93]. The high expression of BMI1 can be achieved by, at least, METT3-mediated N^6^-methyladenosine (m6A) modification of BMI1 mRNA, which promotes BMI1 translation in oral cancer cells [94]. The BMI1 protein can also be stabilized by deubiquitinase USP15 and IL-1 receptor type 2 (IL1R2) in breast CSCs [95].

Moreover, BMI1 can induce EMT and metastasis of cancer cells through cooperating with Twist1, a crucial EMT regulator [96]. In addition, BMI1 is highly expressed in cisplatin-resistant and metastatic HNSCC cells, which exhibit CSC characteristics along with enhanced AP-1 activity and IL-6 signaling as well as the expression of stemness markers, such as aldehyde dehydrogenase (ALDH) and CD44 [89,90,97,98,99,100]. A recent study shows that BMI1 is involved in the RAD51-dependent response to replication stress, which contributes to chemoresistance in breast CSCs [101].

A high expression of BMI1 is associated with poor outcomes in the patients with NPC, glioma, HNSCC, and NSCLC [97,102,103,104,105,106]. The BMI1 expression is higher in metastatic melanoma than in primary cancer, supporting the EMT-promoting activity of BMI1 [107]. Moreover, a high expression of BMI1 is correlated with decreased CD4+/CD8+ T cells in the TME and predicts a poor disease-free survival in patients with breast cancer [108].

### 4.3. G9a

In addition to the PRC complex, several regulators of histone H3K9 methylation, such as the SET domain-containing histone methyltransferases SETDB1 and G9a (also known as euchromatic histone lysine N-methyltransferase 2, EHMT2), play important roles in regulating pluripotency and cancer stemness [109,110]. Importantly, G9a can regulate stemness and tumorigenicity by reprogramming genome-wide DNA methylation in NSCLC [111]. By using human transformed pluripotent cells as a colorectal CSC model, Bergin et al. found that G9a is crucial for the phenotype of embryonic-like transcriptional signatures, such as undifferentiated state, self-renewal, EMT, and tumorigenicity [112]. Additionally, G9a also serves as a functional partner of MYC [113], which is a well-known oncogene and is involved in reprogramming of induced pluripotent stem cell [114]. Liu et al. demonstrated that G9a interacts with Snail to inhibit the expression of E-cadherin, a typical epithelial marker, through H3K9 methylation at the E-cadherin promoter. As a result, G9a promotes EMT and lymph node metastasis in HNSCC [115]. In addition to E-cadherin, G9a also suppresses a pro-inflammatory program to promote breast cancer recurrence. As a result, G9a silences the expression of TNF and inhibits RIPK3-dependent necroptosis; thus, it promotes breast cancer cell survival and relapse [116].

An overexpression of G9a is found in several types of cancer, such as neuroblastoma, ovarian, breast, bladder, and lung cancers, and is usually correlated with poor prognoses [117,118,119,120,121,122]. For example, a high expression of G9a is correlated with shorter overall and relapse-free survival in patients with ovarian and colorectal cancers, respectively [112,121]. High G9a activity is also associated with an increased risk of recurrence in breast cancer [116]. These results indicate that G9a contributes to aggressive cancer phenotypes, which are related to CSC characteristics.

### 4.4. SETDB1

Additionally, SETDB1 is another histone H3K9 methyltransferase and is required for early embryonic development [123]. It regulates the development of neural progenitor cells and contributes to the maintenance of hematopoietic stem and progenitor cells [124,125]. In rapidly renewing intestinal epithelium, SETDB1 is required for intestinal epithelial differentiation and homeostasis [126].

Importantly, SETDB1 contributes to the proliferation and migration of colorectal cancer cells via inhibiting p53 and epigenetically silencing p21 expression [127,128]. Overexpression of SETDB1 suppresses BAX expression and inhibits 5-fluorouracil-induced apoptosis in colorectal cancer cells [128]. These results imply the CSC properties of chemoresistance and EMT for SETDB1. In human cancers, an upregulation of SETDB1 is correlated with unfavorable prognoses in patients with melanoma, NPC, colorectal and several cancers [128,129,130,131].

## 5. Targeting Epigenetic Modifiers to Activate Interferon Response and Suppress CSCs

**Figure 3:**
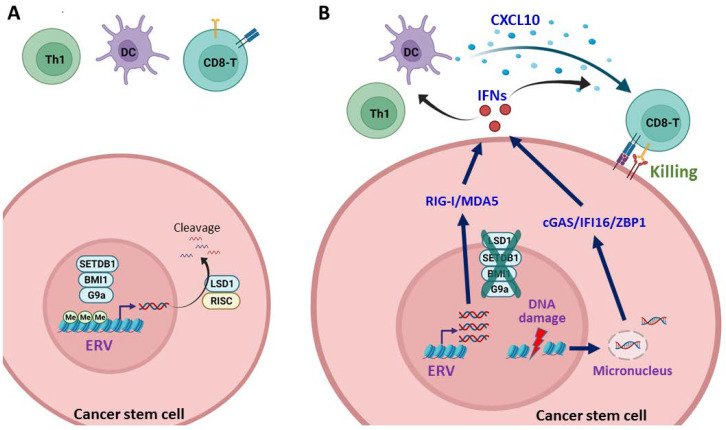
Inhibition of epigenetic modifiers in the induction of interferon response and recruitment of CD8+ T cells. (**A**) The histone modifiers LSD1, SETDB1, BMI1, and G9a are overexpressed in cancer stem cells (CSCs), leading to silencing of endogenous retrovirus (ERV) by the hypermethylation and degradation of cytosolic RNA by the RNA-induced silencing complex (RISC). As a result, the interferon response is suppressed and the CSCs are kept away from the killing of cytotoxic CD8+ T cells; (**B**) Inhibition of LSD1, SETDB1, BMI1, or G9a results in de-repression of ERV and induction of interferons (IFNs) through RIG-I/MDA5. Besides, inhibition of these epigenetic modifiers causes DNA damage and increases micronucleus formation, leading to elevation of cytosolic DNA and induction of IFNs through cGAS/IFI16/ZBP1. The secreted IFNs activate dendritic cells (DC) and type 1 T helper cells (Th1), which produce the T-cell attractive chemokine CXCL10 to recruit CD8+ T cells for the killing of CSCs. Me, methylation. Graph created with Biorender.com.

Because LSD1, BMI1, G9a, and SETDB1 are enriched in CSCs and are crucial for CSC characteristics, targeting these histone methylation modifiers can be used as a strategy to inhibit CSCs. Another rationale for targeting these epigenetic modifiers in CSCs is to re-activate ERV elements and induce DNA damage, both can trigger interferon response and sculpt a “hot” TME for ICB therapy (Figure 3). The expression of ERV and DNA damage-induced neoantigens may also increase immunogenicity. Moreover, interferon response is essential to maintain CSC dormancy, which prevents cancer relapse and the outgrowth of DTCs and TRCs at distant organs [5,8,132]. Therefore, targeting epigenetic modifiers is a potential strategy by which to enhance the efficacy of cancer immunotherapy [20,23,133].

### 5.1. Targeting LSD1

As is known, LSD1 plays an important role in suppressing ERV elements in mouse embryonic stem cells (mESCs) and in human cancer cells [21,134]. The inhibition of LSD1 activates the expression of ERV elements and type I interferons, which induce antitumor T-cell immunity and sensitize ICB-refractory cancers to ICIs [21,135]. In addition, Mosammaparast et al. show that the knockdown of LSD1 impairs the recruitment of 53BP1 and BRCA1 to the DNA damage sites and represses DNA repair [136]; thus, the amounts of cytosolic DNA may be elevated. The inhibition of LSD1 can also promote the differentiation of conventional DCs, which are crucial for antitumor immunosurveillance and ICB therapy [137]. In the mouse xenograft model of TNBC, LSD1 inhibitors in combination with the PD-1 antibody significantly suppress tumor growth and pulmonary metastasis. Moreover, increased CD8+ T cell infiltration is observed in the xenograft tumors [138]. Targeting LSD1 also contributes to an enhanced efficacy of ICB therapy in mouse models of HNSCC and cervical cancer [139,140], as well as in an organoid model of ovarian cancer [135]. Moreover, LSD1 ablation can enhance anti-tumor activity of CD19 CAR-T cells [141].

By performing an analysis of TCGA data sets, the expression of LSD1 is conversely correlated with CD8+ T cell infiltration in various types of cancer, and high LSD1 expression is a predictor of poor prognosis for HNSCC patients [21,140]. Qin et al. also reported that the expressions of T-cell attraction chemokines CXCL9 and CXCL10 are inversely correlated with that of LSD1 in TNBC [138]. These results indicate that the anti-tumor activity of LSD1 inhibition is correlated with the activation and infiltration of CD8+ T cells, which is attributed to the de-repression of ERV elements and induction of interferon response. Thus, LSD1 inhibition in combination with ICB may improve cancer treatment. However, LSD1 ablation may induce the expression of TGF-β that negatively regulates T-cell immunity. Sheng demonstrated that the concurrent ablation of LSD1 and TGF-β in combination with ICB therapy is helpful for the eradication of poorly immunogenic tumors and also for the protection from tumor relapse. Thus, a triple combination of LSD1, TGF-β, and immune checkpoint inhibitors may be required for the treatment of ICB-refractory cancers [142].

In addition to augmenting antitumor immunity, LSD1 ablation can suppress the stem cell-like properties of HNSCC, HCC, glioma, small cell lung cancer, and breast cancers of luminal-B, HER2-positive, and TNBC subtypes through attenuating Wnt/β-catenin, Notch, BMI1, and SOX2-deriven stemness signaling. Inhibition of LSD1 also promotes epithelial differentiation through de-repression of fate-determining transcription factors [79,140,143,144,145,146,147]. These results indicate that targeting LSD1 can suppress CSC characteristics and enhance the efficacy of ICB therapy [22,74]. Some LSD1 inhibitors are currently subject to clinical trials [22,148].

### 5.2. Targeting BMI1

Importantly, BMI1 plays a critical role in the self-renewal and chemoresistance of HNSCC CSCs [89,90]. The inhibition of BMI1 has been shown to suppress sphere formation and decrease resistance to cisplatin and 5-fluorouracil in HNSCC [89,149]. Additionally, BMI1 depletion can impair DNA double-strand break (DSB) repair [150]. Furthermore, inhibition of BMI1 in HNSCC cells can activate interferon response via cGAS-STING axis and elevate the secretion of T-cell-attracting chemokines including CXCL9, CXCL10, and CXCL11. As a result, the inhibition of BMI1 increases tumor infiltration of CD8+ T cells, improves the efficacy of ICB therapy, and prevents tumor metastasis and relapse in a HNSCC mouse model [151]. The inhibition of BMI1 also suppresses CSC characteristics and tumor growth in colorectal cancer [152,153], breast cancer [154], HCC [155,156], glioblastoma and neuroblastoma [92,157].

### 5.3. Targeting G9a

As previously mentioned, G9a contributes to CSC characteristics and cancer progression, suggesting that targeting G9a may be a potential strategy to suppress CSCs [109]. It has been shown that G9a ablation impedes DSB repair in a p53-independent manner and sensitizes cancer cells to DNA damaging agents [158]. The inhibition of G9a can enhance IFN-γ-stimulated expression of CXCL9 and CXCL10, which are crucial Th1 chemokines for the recruitment of cytotoxic T cells to the TME of neuroblastoma [159]. In melanoma, G9a inhibition enhances the efficacy of ICB therapy and induces melanoma cell death [160,161]. Bellamy et al. also reported that G9a depletion preferentially triggers apoptosis in neuroblastoma cells with MYCN amplification [118]. The inhibition of G9a in colorectal CSCs induces cell differentiation and suppresses tumor-initiating activity in patient-derived colorectal tumors [112]. In breast cancer, G9a ablation induces TNF and necroptosis, thereby suppressing cancer relapse [116]. The combined inhibition of G9a and EZH2 induces the expression of ERV elements and activates interferon response in multiple myeloma cells, leading to cell cycle arrest and apoptosis [162]. The dual inhibition of G9a and DNA methyltransferase (DNMT) leads to increased tumor infiltrations of NK and CD8+ T cells, enhanced responses to PD-L1 blockade, and facilitated tumor regression in a mouse model of bladder cancer. In human bladder cancer, the levels of G9a and DNMT are correlated with responses to anti-PD-1 immunotherapy [119]. The concurrent inhibition of G9a and class I histone deacetylases (HDACs) suppresses the gene signatures of CSCs, EMT, drug resistance and cell fate determination in breast cancer, resulting in growth suppression and the apoptosis of breast cancer cells [117].

### 5.4. Targeting SETDB1

As SETDB1 is required for 53BP1 repositioning and BRCA1 function during DNA homologous recombination repair, the depletion of SETDB1 increases DNA strand breaks in G2 phase of the cell cycle [163]. The inhibition of SETDB1 also results in the significant activation of ERV elements in ESCs [27,164]. In a mouse model of melanoma, SETDB1-knockout leads to the re-expression of ERV antigens, which are presented by MHC-I on the cell surface, and induces specific cytotoxic T-cell response [165]. In human cancers, the expression of SETDB1 is inversely correlated with several hallmark gene signatures related to immune response. In patients with renal cell carcinoma who received PD-1 blockade therapy, the amplification of SETDB1 gene was found to be associated with poor overall survival [165]. Another study demonstrates that the H3K4 demethylase KDM5B can recruit SETDB1 to ERV elements and repress their expression. The depletion of KDM5B causes the de-repression of ERV elements and induces interferon response, leading to tumor regression and induction of adaptive immune memory [166]. In the tumors of melanoma patients before receiving immunotherapy, the expression of KDM5D is lower in patients with complete response than in those with progressive disease [166]. These results suggest that SETDB1 and KDM5B serve as epigenetic checkpoints to suppress the tumor-intrinsic immune response, and thus can be candidate therapeutic targets for improving immunotherapy.

## 6. Additional Considerations beyond Targeting LSD1, BMI1, G9a, and SETDB1

In addition to LSD1, BMI1, G9a, and SETDB1, other epigenetic modifiers are also involved in the regulation of CSC properties and can be candidate therapeutic targets. For example, targeting EZH2 can de-repress ERV and induce interferon response in Ewing sarcoma [167]. In prostate cancer, EZH2 ablation also triggers an endogenous double-stranded RNA-STING axis-induced interferon response that enhances ICB therapy by increasing antigen presentation, Th1 chemokine signaling, and tumor infiltrations of activated CD8+ T cells and M1 tumor-associated macrophages [168]. Moreover, DNMTs, HDACs, and the poly (ADP-ribose) polymerases, which cause DNA PARylation upon DNA damage, are also promising targets to induce cytosolic nucleic acids via DNA damage and ERV re-expression. Consequently, the interferon response is triggered and the efficacy of ICB therapy is enhanced [29,37,133,169,170].

Although targeting epigenetic modifiers is an effective strategy by which to boost the antitumor immune response for CSC eradication in many preclinical studies, its application in clinics is still challenged. Because many epigenetic modifiers are essential for the functions and homeostasis of normal stem/progenitor cells, the depletion of these epigenetic modifiers may cause severe adverse effects to the healthy tissues and organs [74,87,126,171]. Thus, a strategy that can specifically deliver the epigenetic modifier inhibitors to the TME but not to other healthy tissues needs to be adapted.

## 7. Conclusions

The histone methylation modifiers LSD1, BMI1, G9a, and SETDB1 are crucial for CSC characteristics, such as self-renewal, differentiation, homeostasis, EMT, chemoresistance, and immune evasion. Their expressions are usually higher in CSCs than in bulk tumor cells. Therefore, these epigenetic modifiers can be used as CSC biomarkers and therapeutic targets.

The inhibition of LSD1, BMI1, G9a, or SETDB1 can de-repress ERV elements and induce DNA damage, and both events elicit cytosolic nucleic acids and trigger an interferon response. The induced interferons not only inhibit cancer cells but also activate innate and adaptive immune responses. For example, interferons can activate DC and NK cells to ensure immunosurveillance and prime Th1 and CD8+ T cells for tumor killing. Thus, the activation of the interferon response can contribute to the “hot” TME and improve ICB therapy. Moreover, interferons play a critical role in CSC dormancy and, thus, prevent cancer relapse. Nevertheless, a continued hyperactivation of interferon response may cause tissue damage and autoimmune disease. Therefore, further investigations that unveil the precise regulation of interferon response are required for future clinical applications in anticancer therapy.

## Data Availability

Not applicable.

## References

[1] Batlle E., Clevers H. (2017). Cancer stem cells revisited. Nat. Med..

[2] Saito S., Lin Y.C., Nakamura Y., Eckner R., Wuputra K., Kuo K.K., Lin C.S., Yokoyama K.K. (2019). Potential application of cell reprogramming techniques for cancer research. Cell. Mol. Life Sci. CMLS.

[3] Chu P.Y., Hou M.F., Lai J.C., Chen L.F., Lin C.S. (2019). Cell reprogramming in tumorigenesis and its therapeutic implications for breast cancer. Int. J. Mol. Sci..

[4] Keyvani-Ghamsari S., Khorsandi K., Rasul A., Zaman M.K. (2021). Current understanding of epigenetics mechanism as a novel target in reducing cancer stem cells resistance. Clin. Epigenetics.

[5] Sistigu A., Musella M., Galassi C., Vitale I., De Maria R. (2020). Tuning cancer fate: Tumor microenvironment’s role in cancer stem cell quiescence and reawakening. Front. Immunol..

[6] Saygin C., Matei D., Majeti R., Reizes O., Lathia J.D. (2019). Targeting cancer stemness in the clinic: From hype to hope. Cell Stem Cell.

[7] Qin S., Jiang J., Lu Y., Nice E.C., Huang C., Zhang J., He W. (2020). Emerging role of tumor cell plasticity in modifying therapeutic response. Signal Transduct. Target. Ther..

[8] Chernosky N.M., Tamagno I. (2021). The role of the innate immune system in cancer dormancy and relapse. Cancers.

[9] Tsuchiya H., Shiota G. (2021). Immune evasion by cancer stem cells. Regen. Ther..

[10] Lei M.M.L., Lee T.K.W. (2021). Cancer stem cells: Emerging key players in immune evasion of cancers. Front. Cell Dev. Biol..

[11] Galassi C., Musella M., Manduca N., Maccafeo E., Sistigu A. (2021). The immune privilege of cancer stem cells: A key to understanding tumor immune escape and therapy failure. Cells.

[12] Morrison B.J., Steel J.C., Morris J.C. (2018). Reduction of MHC-I expression limits T-lymphocyte-mediated killing of cancer-initiating cells. BMC Cancer.

[13] Tallerico R., Garofalo C., Carbone E. (2016). A new biological feature of natural killer cells: The recognition of solid tumor-derived cancer stem cells. Front. Immunol..

[14] Lee Y., Shin J.H., Longmire M., Wang H., Kohrt H.E., Chang H.Y., Sunwoo J.B. (2016). CD44+ cells in head and neck squamous cell carcinoma suppress T-cell-mediated immunity by selective constitutive and inducible expression of PD-L1. Clin. Cancer Res..

[15] Sharma P., Hu-Lieskovan S., Wargo J.A., Ribas A. (2017). Primary, adaptive, and acquired resistance to cancer immunotherapy. Cell.

[16] Wainwright E.N., Scaffidi P. (2017). Epigenetics and cancer stem cells: Unleashing, hijacking, and restricting cellular plasticity. Trends Cancer.

[17] Toh T.B., Lim J.J., Chow E.K. (2017). Epigenetics in cancer stem cells. Mol. Cancer.

[18] Wu D.C., Wang S.S.W., Liu C.J., Wuputra K., Kato K., Lee Y.L., Lin Y.C., Tsai M.H., Ku C.C., Lin W.H. (2017). Reprogramming antagonizes the oncogenicity of HOXA13-long noncoding RNA HOTTIP axis in gastric cancer cells. Stem Cells.

[19] Topper M.J., Vaz M., Marrone K.A., Brahmer J.R., Baylin S.B. (2020). The emerging role of epigenetic therapeutics in immuno-oncology. Nat. Rev. Clin. Oncol..

[20] Galassi C., Vitale I., Galluzzi L. (2021). Using epigenetic modifiers to target cancer stem cell immunoevasion. Cancer Cell.

[21] Sheng W., LaFleur M.W., Nguyen T.H., Chen S., Chakravarthy A., Conway J.R., Li Y., Chen H., Yang H., Hsu P.H. (2018). LSD1 ablation stimulates anti-tumor immunity and enables checkpoint blockade. Cell.

[22] Karakaidos P., Verigos J., Magklara A. (2019). LSD1/KDM1A, a gate-keeper of cancer stemness and a promising therapeutic target. Cancers.

[23] Liu Z., Ren Y., Weng S., Xu H., Li L., Han X. (2022). A new trend in cancer treatment: The combination of epigenetics and immunotherapy. Front. Immunol..

[24] Jansz N., Faulkner G.J. (2021). Endogenous retroviruses in the origins and treatment of cancer. Genome Biol..

[25] Geis F.K., Goff S.P. (2020). Silencing and transcriptional regulation of endogenous retroviruses: An overview. Viruses.

[26] Groh S., Schotta G. (2017). Silencing of endogenous retroviruses by heterochromatin. Cell. Mol. Life Sci. CMLS.

[27] Matsui T., Leung D., Miyashita H., Maksakova I.A., Miyachi H., Kimura H., Tachibana M., Lorincz M.C., Shinkai Y. (2010). Proviral silencing in embryonic stem cells requires the histone methyltransferase ESET. Nature.

[28] Canadas I., Thummalapalli R., Kim J.W., Kitajima S., Jenkins R.W., Christensen C.L., Campisi M., Kuang Y., Zhang Y., Gjini E. (2018). Tumor innate immunity primed by specific interferon-stimulated endogenous retroviruses. Nat. Med..

[29] Hogg S.J., Beavis P.A., Dawson M.A., Johnstone R.W. (2020). Targeting the epigenetic regulation of antitumour immunity. Nat. Rev. Drug Discov..

[30] von Locquenghien M., Rozalen C., Celia-Terrassa T. (2021). Interferons in cancer immunoediting: Sculpting metastasis and immunotherapy response. J. Clin. Investig..

[31] Fenton S.E., Saleiro D., Platanias L.C. (2021). Type I and II interferons in the anti-tumor immune response. Cancers.

[32] Martin-Hijano L., Sainz B. (2020). The interactions between cancer stem cells and the innate interferon signaling pathway. Front. Immunol..

[33] Lind N.A., Rael V.E., Pestal K., Liu B., Barton G.M. (2021). Regulation of the nucleic acid-sensing toll-like receptors. Nat. Rev. Immunol.

[34] Cinat D., Coppes R.P., Barazzuol L. (2021). DNA damage-induced inflammatory microenvironment and adult stem cell response. Front. Cell Dev. Biol..

[35] Vanpouille-Box C., Demaria S., Formenti S.C., Galluzzi L. (2018). Cytosolic DNA sensing in organismal tumor control. Cancer Cell.

[36] Kwon J., Bakhoum S.F. (2020). The cytosolic DNA-sensing cGAS-STING pathway in cancer. Cancer Discov..

[37] Huang J.L., Chang Y.T., Hong Z.Y., Lin C.S. (2022). Targeting DNA damage response and immune checkpoint for anticancer therapy. Int. J. Mol. Sci..

[38] Schoggins J.W. (2019). Interferon-stimulated genes: What do they all do?. Annu. Rev. Virol..

[39] Karin N. (2020). CXCR3 ligands in cancer and autoimmunity, chemoattraction of effector T cells, and beyond. Front. Immunol..

[40] Huang Y.-C., Huang J.-L., Tseng L.-C., Yu P.-H., Chen S.-Y., Lin C.-S. (2022). High expression of interferon pathway genes CXCL10 and STAT2 is associated with activated T-cell signature and better outcome of oral cancer patients. J. Pers. Med..

[41] Zitvogel L., Galluzzi L., Kepp O., Smyth M.J., Kroemer G. (2015). Type I interferons in anticancer immunity. Nat. Rev. Immunol..

[42] Hartlova A., Erttmann S.F., Raffi F.A., Schmalz A.M., Resch U., Anugula S., Lienenklaus S., Nilsson L.M., Kroger A., Nilsson J.A. (2015). DNA damage primes the type I interferon system via the cytosolic DNA sensor STING to promote anti-microbial innate immunity. Immunity.

[43] Doherty M.R., Cheon H., Junk D.J., Vinayak S., Varadan V., Telli M.L., Ford J.M., Stark G.R., Jackson M.W. (2017). Interferon-beta represses cancer stem cell properties in triple-negative breast cancer. Proc. Natl. Acad. Sci. USA.

[44] Doherty M.R., Parvani J.G., Tamagno I., Junk D.J., Bryson B.L., Cheon H.J., Stark G.R., Jackson M.W. (2019). The opposing effects of interferon-beta and oncostatin-M as regulators of cancer stem cell plasticity in triple-negative breast cancer. Breast Cancer Res. BCR.

[45] Zhuang X., Shi G., Hu X., Wang H., Sun W., Wu Y. (2021). Interferon-gamma inhibits aldehyde dehydrogenasebright cancer stem cells in the 4T1 mouse model of breast cancer. Chin. Med. J..

[46] Buoncervello M., Romagnoli G., Buccarelli M., Fragale A., Toschi E., Parlato S., Lucchetti D., Macchia D., Spada M., Canini I. (2016). IFN-alpha potentiates the direct and immune-mediated antitumor effects of epigenetic drugs on both metastatic and stem cells of colorectal cancer. Oncotarget.

[47] Du Z., Cai C., Sims M., Boop F.A., Davidoff A.M., Pfeffer L.M. (2017). The effects of type I interferon on glioblastoma cancer stem cells. Biochem. Biophys. Res. Commun..

[48] Liu Y., Liang X., Yin X., Lv J., Tang K., Ma J., Ji T., Zhang H., Dong W., Jin X. (2017). Blockade of IDO-kynurenine-AhR metabolic circuitry abrogates IFN-gamma-induced immunologic dormancy of tumor-repopulating cells. Nat. Commun..

[49] Liu Y., Lv J., Liu J., Liang X., Jin X., Xie J., Zhang L., Chen D., Fiskesund R., Tang K. (2018). STAT3/p53 pathway activation disrupts IFN-beta-induced dormancy in tumor-repopulating cells. J. Clin. Investig..

[50] Lan Q., Peyvandi S., Duffey N., Huang Y.T., Barras D., Held W., Richard F., Delorenzi M., Sotiriou C., Desmedt C. (2019). Type I interferon/IRF7 axis instigates chemotherapy-induced immunological dormancy in breast cancer. Oncogene.

[51] Owen K.L., Gearing L.J., Zanker D.J., Brockwell N.K., Khoo W.H., Roden D.L., Cmero M., Mangiola S., Hong M.K., Spurling A.J. (2020). Prostate cancer cell-intrinsic interferon signaling regulates dormancy and metastatic outgrowth in bone. EMBO Rep..

[52] Correia A.L., Guimaraes J.C., Auf der Maur P., De Silva D., Trefny M.P., Okamoto R., Bruno S., Schmidt A., Mertz K., Volkmann K. (2021). Hepatic stellate cells suppress NK cell-sustained breast cancer dormancy. Nature.

[53] Wang J.M., Liu B.Q., Zhang Q., Hao L., Li C., Yan J., Zhao F.Y., Qiao H.Y., Jiang J.Y., Wang H.Q. (2020). ISG15 suppresses translation of ABCC2 via ISGylation of hnRNPA2B1 and enhances drug sensitivity in cisplatin resistant ovarian cancer cells. Biochim. Biophys. Acta. Mol. Cell Res..

[54] Zhang Q., Wang J., Qiao H., Huyan L., Liu B., Li C., Jiang J., Zhao F., Wang H., Yan J. (2021). ISG15 is downregulated by KLF12 and implicated in maintenance of cancer stem cell-like features in cisplatin-resistant ovarian cancer. J. Cell. Mol. Med..

[55] Chen R.H., Du Y., Han P., Wang H.B., Liang F.Y., Feng G.K., Zhou A.J., Cai M.Y., Zhong Q., Zeng M.S. (2016). ISG15 predicts poor prognosis and promotes cancer stem cell phenotype in nasopharyngeal carcinoma. Oncotarget.

[56] Xu L., Huang T.J., Hu H., Wang M.Y., Shi S.M., Yang Q., Lin F., Qiang Y.Y., Mei Y., Lang Y.H. (2018). The developmental transcription factor IRF6 attenuates ABCG2 gene expression and distinctively reverses stemness phenotype in nasopharyngeal carcinoma. Cancer Lett..

[57] Li Z., Yang W., Qiu J., Xu H., Fan B., Li K., Zhou J., Li Y. (2021). Decreased interferon regulatory factor 6 expression due to DNA hypermethylation predicts an unfavorable prognosis in clear cell renal cell carcinoma. J. Cancer.

[58] Huang W.C., Tung S.L., Chen Y.L., Chen P.M., Chu P.Y. (2018). IFI44L is a novel tumor suppressor in human hepatocellular carcinoma affecting cancer stemness, metastasis, and drug resistance via regulating met/Src signaling pathway. BMC cancer.

[59] Zhu Y., Karakhanova S., Huang X., Deng S.P., Werner J., Bazhin A.V. (2014). Influence of interferon-alpha on the expression of the cancer stem cell markers in pancreatic carcinoma cells. Exp. Cell Res..

[60] Sainz B., Martin B., Tatari M., Heeschen C., Guerra S. (2014). ISG15 is a critical microenvironmental factor for pancreatic cancer stem cells. Cancer Res..

[61] Gross E.T.E., Peinado C.D., Jung Y., Han S., Liu B., Santosa E.K., Bui J.D. (2019). Identification and editing of stem-like cells in methylcholanthrene-induced sarcomas. Oncoimmunology.

[62] Arico E., Castiello L., Capone I., Gabriele L., Belardelli F. (2019). Type I Interferons and Cancer: An evolving story demanding novel clinical applications. Cancers.

[63] Song M., Ping Y., Zhang K., Yang L., Li F., Zhang C., Cheng S., Yue D., Maimela N.R., Qu J. (2019). Low-dose IFNgamma induces tumor cell stemness in tumor microenvironment of non-small cell lung cancer. Cancer Res..

[64] Castiello L., Sestili P., Schiavoni G., Dattilo R., Monque D.M., Ciaffoni F., Iezzi M., Lamolinara A., Sistigu A., Moschella F. (2018). Disruption of IFN-I signaling promotes HER2/Neu tumor progression and breast cancer stem cells. Cancer Immunol. Res..

[65] Li J., Chen J.N., Zeng T.T., He F., Chen S.P., Ma S., Bi J., Zhu X.F., Guan X.Y. (2016). CD133+ liver cancer stem cells resist interferon-gamma-induced autophagy. BMC Cancer.

[66] Shirasaki T., Honda M., Yamashita T., Nio K., Shimakami T., Shimizu R., Nakasyo S., Murai K., Shirasaki N., Okada H. (2018). The osteopontin-CD44 axis in hepatic cancer stem cells regulates IFN signaling and HCV replication. Sci. Rep..

[67] Pezze L., Meskyte E.M., Forcato M., Pontalti S., Badowska K.A., Rizzotto D., Skvortsova I.-I., Bicciato S., Ciribilli Y. (2021). ETV7 regulates breast cancer stem-like cell features by repressing IFN-response genes. Cell Death Dis..

[68] Zhan X., Guo S., Li Y., Ran H., Huang H., Mi L., Wu J., Wang X., Xiao D., Chen L. (2020). Glioma stem-like cells evade interferon suppression through MBD3/NuRD complex-mediated STAT1 downregulation. J. Exp. Med..

[69] Siddique H.R., Saleem M. (2012). Role of BMI1, a stem cell factor, in cancer recurrence and chemoresistance: Preclinical and clinical evidences. Stem Cells.

[70] Wang H., Gao L., Qi M., Su P., Xiong X., Zhao J., Hu J., Han B. (2021). BTF3 promotes stemness and inhibits typeinterferon signaling pathway in triple-negative breast cancer. Biochem. Biophys. Res. Commun..

[71] Hu J., Sun F., Chen W., Zhang J., Zhang T., Qi M., Feng T., Liu H., Li X., Xing Y. (2019). BTF3 sustains cancer stem-like phenotype of prostate cancer via stabilization of BMI1. J. Exp. Clin. Cancer Res. CR.

[72] Zhou W., Yun Z., Wang T., Li C., Zhang J. (2021). BTF3-mediated regulation of BMI1 promotes colorectal cancer through influencing epithelial-mesenchymal transition and stem cell-like traits. Int. J. Biol. Macromol..

[73] Celia-Terrassa T., Liu D.D., Choudhury A., Hang X., Wei Y., Zamalloa J., Alfaro-Aco R., Chakrabarti R., Jiang Y.Z., Koh B.I. (2017). Normal and cancerous mammary stem cells evade interferon-induced constraint through the miR-199a-LCOR axis. Nat. Cell Biol..

[74] Martinez-Gamero C., Malla S., Aguilo F. (2021). LSD1: Expanding functions in stem Cells and differentiation. Cells.

[75] Dan S., Song Y., Duan X., Pan X., Chen C., She S., Su T., Li J., Chen X., Zhou Y. (2021). LSD1-mediated demethylation of OCT4 safeguards pluripotent stem cells by maintaining the transcription of PORE-motif-containing genes. Sci. Rep..

[76] Wang J., Hevi S., Kurash J.K., Lei H., Gay F., Bajko J., Su H., Sun W., Chang H., Xu G. (2009). The lysine demethylase LSD1 (KDM1) is required for maintenance of global DNA methylation. Nat. Genet..

[77] Amente S., Lania L., Majello B. (2013). The histone LSD1 demethylase in stemness and cancer transcription programs. Biochim. Biophys. Acta.

[78] Verigos J., Karakaidos P., Kordias D., Papoudou-Bai A., Evangelou Z., Harissis H.V., Klinakis A., Magklara A. (2019). The histone demethylase LSD1/KappaDM1A mediates chemoresistance in breast cancer via regulation of a stem cell program. Cancers.

[79] Huang M., Chen C., Geng J., Han D., Wang T., Xie T., Wang L., Wang Y., Wang C., Lei Z. (2017). Targeting KDM1A attenuates Wnt/beta-catenin signaling pathway to eliminate sorafenib-resistant stem-like cells in hepatocellular carcinoma. Cancer Lett..

[80] Boulding T., McCuaig R.D., Tan A., Hardy K., Wu F., Dunn J., Kalimutho M., Sutton C.R., Forwood J.K., Bert A.G. (2018). LSD1 activation promotes inducible EMT programs and modulates the tumour microenvironment in breast cancer. Sci. Rep..

[81] Cho H.S., Suzuki T., Dohmae N., Hayami S., Unoki M., Yoshimatsu M., Toyokawa G., Takawa M., Chen T., Kurash J.K. (2011). Demethylation of RB regulator MYPT1 by histone demethylase LSD1 promotes cell cycle progression in cancer cells. Cancer Res..

[82] Huang J., Sengupta R., Espejo A.B., Lee M.G., Dorsey J.A., Richter M., Opravil S., Shiekhattar R., Bedford M.T., Jenuwein T. (2007). p53 is regulated by the lysine demethylase LSD1. Nature.

[83] Majello B., Gorini F., Sacca C.D., Amente S. (2019). Expanding the role of the histone lysine-specific demethylase LSD1 in cancer. Cancers.

[84] Hosseini A., Minucci S. (2017). A comprehensive review of lysine-specific demethylase 1 and its roles in cancer. Epigenomics.

[85] Zhao L.J., Li Y.Y., Zhang Y.T., Fan Q.Q., Ren H.M., Zhang C., Mardinoglu A., Chen W.C., Pang J.R., Shen D.D. (2021). Lysine demethylase LSD1 delivered via small extracellular vesicles promotes gastric cancer cell stemness. EMBO Rep..

[86] Wang H., Wang L., Erdjument-Bromage H., Vidal M., Tempst P., Jones R.S., Zhang Y. (2004). Role of histone H2A ubiquitination in Polycomb silencing. Nature.

[87] Park I.K., Qian D., Kiel M., Becker M.W., Pihalja M., Weissman I.L., Morrison S.J., Clarke M.F. (2003). Bmi-1 is required for maintenance of adult self-renewing haematopoietic stem cells. Nature.

[88] Molofsky A.V., Pardal R., Iwashita T., Park I.K., Clarke M.F., Morrison S.J. (2003). Bmi-1 dependence distinguishes neural stem cell self-renewal from progenitor proliferation. Nature.

[89] Chen D., Wu M., Li Y., Chang I., Yuan Q., Ekimyan-Salvo M., Deng P., Yu B., Yu Y., Dong J. (2017). Targeting BMI1(+) cancer stem cells overcomes chemoresistance and inhibits metastases in squamous cell carcinoma. Cell Stem. Cell..

[90] Prince M.E., Sivanandan R., Kaczorowski A., Wolf G.T., Kaplan M.J., Dalerba P., Weissman I.L., Clarke M.F., Ailles L.E. (2007). Identification of a subpopulation of cells with cancer stem cell properties in head and neck squamous cell carcinoma. Proc. Natl. Acad. Sci. USA.

[91] Chiba T., Miyagi S., Saraya A., Aoki R., Seki A., Morita Y., Yonemitsu Y., Yokosuka O., Taniguchi H., Nakauchi H. (2008). The polycomb gene product BMI1 contributes to the maintenance of tumor-initiating side population cells in hepatocellular carcinoma. Cancer Res..

[92] Cui H., Hu B., Li T., Ma J., Alam G., Gunning W.T., Ding H.F. (2007). Bmi-1 is essential for the tumorigenicity of neuroblastoma cells. Am. J. Pathol..

[93] Vora P., Seyfrid M., Venugopal C., Qazi M.A., Salim S., Isserlin R., Subapanditha M., O’Farrell E., Mahendram S., Singh M. (2019). Bmi1 regulates human glioblastoma stem cells through activation of differential gene networks in CD133+ brain tumor initiating cells. J. Neuro-Oncol..

[94] Liu L., Wu Y., Li Q., Liang J., He Q., Zhao L., Chen J., Cheng M., Huang Z., Ren H. (2020). METTL3 promotes tumorigenesis and metastasis through BMI1 m(6)A methylation in oral squamous cell carcinoma. Mol. Ther. J. Am. Soc. Gene Ther..

[95] Zhang L., Qiang J., Yang X., Wang D., Rehman A.U., He X., Chen W., Sheng D., Zhou L., Jiang Y.Z. (2020). IL1R2 blockade suppresses breast tumorigenesis and progression by impairing USP15-dependent BMI1 stability. Adv. Sci..

[96] Yang M.H., Hsu D.S., Wang H.W., Wang H.J., Lan H.Y., Yang W.H., Huang C.H., Kao S.Y., Tzeng C.H., Tai S.K. (2010). Bmi1 is essential in Twist1-induced epithelial-mesenchymal transition. Nat. Cell Biol..

[97] Herzog A.E., Warner K.A., Zhang Z., Bellile E., Bhagat M.A., Castilho R.M., Wolf G.T., Polverini P.J., Pearson A.T., Nor J.E. (2021). The IL-6R and Bmi-1 axis controls self-renewal and chemoresistance of head and neck cancer stem cells. Cell Death Dis..

[98] Kim H.S., Chen Y.C., Nor F., Warner K.A., Andrews A., Wagner V.P., Zhang Z., Zhang Z., Martins M.D., Pearson A.T. (2017). Endothelial-derived interleukin-6 induces cancer stem cell motility by generating a chemotactic gradient towards blood vessels. Oncotarget.

[99] Nor C., Zhang Z., Warner K.A., Bernardi L., Visioli F., Helman J.I., Roesler R., Nor J.E. (2014). Cisplatin induces Bmi-1 and enhances the stem cell fraction in head and neck cancer. Neoplasia.

[100] Krishnamurthy S., Warner K.A., Dong Z., Imai A., Nor C., Ward B.B., Helman J.I., Taichman R.S., Bellile E.L., McCauley L.K. (2014). Endothelial interleukin-6 defines the tumorigenic potential of primary human cancer stem cells. Stem. Cells.

[101] Azzoni V., Wicinski J., Macario M., Castagne M., Finetti P., Ambrosova K., Rouault C.D., Serge A., Farina A., Agavnian E. (2022). BMI1 nuclear location is critical for RAD51-dependent response to replication stress and drives chemoresistance in breast cancer stem cells. Cell Death Dis..

[102] Huber G.F., Albinger-Hegyi A., Soltermann A., Roessle M., Graf N., Haerle S.K., Holzmann D., Moch H., Hegyi I. (2011). Expression patterns of Bmi-1 and p16 significantly correlate with overall, disease-specific, and recurrence-free survival in oropharyngeal squamous cell carcinoma. Cancer.

[103] Li J., Gong L.Y., Song L.B., Jiang L.L., Liu L.P., Wu J., Yuan J., Cai J.C., He M., Wang L. (2010). Oncoprotein Bmi-1 renders apoptotic resistance to glioma cells through activation of the IKK-nuclear factor-kappaB Pathway. Am. J. Pathol..

[104] Hayry V., Makinen L.K., Atula T., Sariola H., Makitie A., Leivo I., Keski-Santti H., Lundin J., Haglund C., Hagstrom J. (2010). Bmi-1 expression predicts prognosis in squamous cell carcinoma of the tongue. Br. J. Cancer.

[105] Vrzalikova K., Skarda J., Ehrmann J., Murray P.G., Fridman E., Kopolovic J., Knizetova P., Hajduch M., Klein J., Kolek V. (2008). Prognostic value of Bmi-1 oncoprotein expression in NSCLC patients: A tissue microarray study. J. Cancer Res. Clin. Oncol..

[106] Song L.B., Zeng M.S., Liao W.T., Zhang L., Mo H.Y., Liu W.L., Shao J.Y., Wu Q.L., Li M.Z., Xia Y.F. (2006). Bmi-1 is a novel molecular marker of nasopharyngeal carcinoma progression and immortalizes primary human nasopharyngeal epithelial cells. Cancer Res..

[107] Mihic-Probst D., Kuster A., Kilgus S., Bode-Lesniewska B., Ingold-Heppner B., Leung C., Storz M., Seifert B., Marino S., Schraml P. (2007). Consistent expression of the stem cell renewal factor BMI-1 in primary and metastatic melanoma. Int. J. Cancer.

[108] Chung Y., Min K.W., Kim D.H., Son B.K., Do S.I., Chae S.W., Kwon M.J. (2021). High BMI1 expression with low CD8+ and CD4+ T cell activity could promote breast cancer cell survival: A machine learning approach. J. Pers. Med..

[109] Haebe J.R., Bergin C.J., Sandouka T., Benoit Y.D. (2021). Emerging role of G9a in cancer stemness and promises as a therapeutic target. Oncogenesis.

[110] Lee S., Lee C., Hwang C.Y., Kim D., Han Y., Hong S.N., Kim S.H., Cho K.H. (2020). Network inference analysis identifies SETDB1 as a key regulator for reverting colorectal cancer cells into differentiated normal-Like cells. Mol. Cancer Res. MCR.

[111] Pangeni R.P., Yang L., Zhang K., Wang J., Li W., Guo C., Yun X., Sun T., Wang J., Raz D.J. (2020). G9a regulates tumorigenicity and stemness through genome-wide DNA methylation reprogramming in non-small cell lung cancer. Clin. Epigenetics.

[112] Bergin C.J., Zouggar A., Haebe J.R., Masibag A.N., Desrochers F.M., Reilley S.Y., Agrawal G., Benoit Y.D. (2021). G9a controls pluripotent-like identity and tumor-initiating function in human colorectal cancer. Oncogene.

[113] Tu W.B., Shiah Y.J., Lourenco C., Mullen P.J., Dingar D., Redel C., Tamachi A., Ba-Alawi W., Aman A., Al-Awar R. (2018). MYC interacts with the G9a histone methyltransferase to drive transcriptional repression and tumorigenesis. Cancer Cell.

[114] Takahashi K., Yamanaka S. (2006). Induction of pluripotent stem cells from mouse embryonic and adult fibroblast cultures by defined factors. Cell.

[115] Liu S., Ye D., Guo W., Yu W., He Y., Hu J., Wang Y., Zhang L., Liao Y., Song H. (2015). G9a is essential for EMT-mediated metastasis and maintenance of cancer stem cell-like characters in head and neck squamous cell carcinoma. Oncotarget.

[116] Mabe N.W., Garcia N.M.G., Wolery S.E., Newcomb R., Meingasner R.C., Vilona B.A., Lupo R., Lin C.C., Chi J.T., Alvarez J.V. (2020). G9a promotes breast cancer recurrence through repression of a pro-inflammatory program. Cell Rep..

[117] Lin H.Y., Wu H.J., Chen S.Y., Hou M.F., Lin C.S., Chu P.Y. (2022). Epigenetic therapy combination of UNC0638 and CI-994 suppresses breast cancer via epigenetic remodeling of BIRC5 and GADD45A. Biomed. Pharmacother. Biomed. Pharmacother..

[118] Bellamy J., Szemes M., Melegh Z., Dallosso A., Kollareddy M., Catchpoole D., Malik K. (2020). Increased efficacy of histone methyltransferase G9a inhibitors against MYCN-amplified neuroblastoma. Front. Oncol..

[119] Segovia C., San Jose-Eneriz E., Munera-Maravilla E., Martinez-Fernandez M., Garate L., Miranda E., Vilas-Zornoza A., Lodewijk I., Rubio C., Segrelles C. (2019). Inhibition of a G9a/DNMT network triggers immune-mediated bladder cancer regression. Nat. Med..

[120] Casciello F., Windloch K., Gannon F., Lee J.S. (2015). Functional role of G9a histone methyltransferase in cancer. Front. Immunol..

[121] Hua K.T., Wang M.Y., Chen M.W., Wei L.H., Chen C.K., Ko C.H., Jeng Y.M., Sung P.L., Jan Y.H., Hsiao M. (2014). The H3K9 methyltransferase G9a is a marker of aggressive ovarian cancer that promotes peritoneal metastasis. Mol. Cancer.

[122] Chen M.W., Hua K.T., Kao H.J., Chi C.C., Wei L.H., Johansson G., Shiah S.G., Chen P.S., Jeng Y.M., Cheng T.Y. (2010). H3K9 histone methyltransferase G9a promotes lung cancer invasion and metastasis by silencing the cell adhesion molecule Ep-CAM. Cancer Res..

[123] Dodge J.E., Kang Y.K., Beppu H., Lei H., Li E. (2004). Histone H3-K9 methyltransferase ESET is essential for early development. Mol. Cell Biol..

[124] Koide S., Oshima M., Takubo K., Yamazaki S., Nitta E., Saraya A., Aoyama K., Kato Y., Miyagi S., Nakajima-Takagi Y. (2016). Setdb1 maintains hematopoietic stem and progenitor cells by restricting the ectopic activation of nonhematopoietic genes. Blood.

[125] Tan S.L., Nishi M., Ohtsuka T., Matsui T., Takemoto K., Kamio-Miura A., Aburatani H., Shinkai Y., Kageyama R. (2012). Essential roles of the histone methyltransferase ESET in the epigenetic control of neural progenitor cells during development. Development.

[126] Juznic L., Peuker K., Strigli A., Brosch M., Herrmann A., Hasler R., Koch M., Matthiesen L., Zeissig Y., Loscher B.S. (2021). SETDB1 is required for intestinal epithelial differentiation and the prevention of intestinal inflammation. Gut.

[127] Cao N., Yu Y., Zhu H., Chen M., Chen P., Zhuo M., Mao Y., Li L., Zhao Q., Wu M. (2020). SETDB1 promotes the progression of colorectal cancer via epigenetically silencing p21 expression. Cell Death Dis..

[128] Chen K., Zhang F., Ding J., Liang Y., Zhan Z., Zhan Y., Chen L.H., Ding Y. (2017). Histone methyltransferase SETDB1 promotes the progression of colorectal cancer by inhibiting the expression of TP53. J. Cancer.

[129] Lazaro-Camp V.J., Salari K., Meng X., Yang S. (2021). SETDB1 in cancer: Overexpression and its therapeutic implications. Am. J. Cancer Res..

[130] Orouji E., Federico A., Larribere L., Novak D., Lipka D.B., Assenov Y., Sachindra S., Huser L., Granados K., Gebhardt C. (2019). Histone methyltransferase SETDB1 contributes to melanoma tumorigenesis and serves as a new potential therapeutic target. Int. J. Cancer.

[131] Huang J., Huang W., Liu M., Zhu J., Jiang D., Xiong Y., Zhen Y., Yang D., Chen Z., Peng L. (2018). Enhanced expression of SETDB1 possesses prognostic value and promotes cell proliferation, migration and invasion in nasopharyngeal carcinoma. Oncol. Rep..

[132] Jiang X., Liang L., Chen G., Liu C. (2021). Modulation of immune components on stem cell and dormancy in cancer. Cells.

[133] Jones P.A., Ohtani H., Chakravarthy A., De Carvalho D.D. (2019). Epigenetic therapy in immune-oncology. Nat. Rev. Cancer.

[134] Macfarlan T.S., Gifford W.D., Agarwal S., Driscoll S., Lettieri K., Wang J., Andrews S.E., Franco L., Rosenfeld M.G., Ren B. (2011). Endogenous retroviruses and neighboring genes are coordinately repressed by LSD1/KDM1A. Genes Dev..

[135] Soldi R., Ghosh Halder T., Weston A., Thode T., Drenner K., Lewis R., Kaadige M.R., Srivastava S., Daniel Ampanattu S., Rodriguez Del Villar R. (2020). The novel reversible LSD1 inhibitor SP-2577 promotes anti-tumor immunity in SWItch/Sucrose-NonFermentable (SWI/SNF) complex mutated ovarian cancer. PLoS ONE.

[136] Mosammaparast N., Kim H., Laurent B., Zhao Y., Lim H.J., Majid M.C., Dango S., Luo Y., Hempel K., Sowa M.E. (2013). The histone demethylase LSD1/KDM1A promotes the DNA damage response. J. Cell Biol..

[137] Srivastava P., Tzetzo S.L., Gomez E.C., Eng K.H., Jani Sait S.N., Kuechle J.B., Singh P.K., De Jong K., Wiatrowski K.R., Peresie J. (2020). Inhibition of LSD1 in MDS progenitors restores differentiation of CD141(Hi) conventional dendritic cells. Leukemia.

[138] Qin Y., Vasilatos S.N., Chen L., Wu H., Cao Z., Fu Y., Huang M., Vlad A.M., Lu B., Oesterreich S. (2019). Inhibition of histone lysine-specific demethylase 1 elicits breast tumor immunity and enhances antitumor efficacy of immune checkpoint blockade. Oncogene.

[139] Xu S., Wang X., Yang Y., Li Y., Wu S. (2021). LSD1 silencing contributes to enhanced efficacy of anti-CD47/PD-L1 immunotherapy in cervical cancer. Cell Death Dis..

[140] Han Y., Xu S., Ye W., Wang Y., Zhang X., Deng J., Zhang Z., Liu L., Liu S. (2021). Targeting LSD1 suppresses stem cell-like properties and sensitizes head and neck squamous cell carcinoma to PD-1 blockade. Cell Death Dis..

[141] Zhang J., Zhu J., Zheng G., Wang Q., Li X., Feng Y., Shang F., He S., Jiang Q., Shi B. (2021). Co-expression of miR155 or LSD1 shRNA increases the anti-tumor functions of CD19 CAR-T cells. Front. Immunol..

[142] Sheng W., Liu Y., Chakraborty D., Debo B., Shi Y. (2021). Simultaneous inhibition of LSD1 and TGFbeta enables eradication of poorly immunogenic tumors with anti-PD-1 treatment. Cancer Discov..

[143] Zhou M., Venkata P.P., Viswanadhapalli S., Palacios B., Alejo S., Chen Y., He Y., Pratap U.P., Liu J., Zou Y. (2021). KDM1A inhibition is effective in reducing stemness and treating triple negative breast cancer. Breast Cancer Res. Treat..

[144] Cuyas E., Gumuzio J., Verdura S., Brunet J., Bosch-Barrera J., Martin-Castillo B., Alarcon T., Encinar J.A., Martin A.G., Menendez J.A. (2020). The LSD1 inhibitor iadademstat (ORY-1001) targets SOX2-driven breast cancer stem cells: A potential epigenetic therapy in luminal-B and HER2-positive breast cancer subtypes. Aging.

[145] Egolf S., Aubert Y., Doepner M., Anderson A., Maldonado-Lopez A., Pacella G., Lee J., Ko E.K., Zou J., Lan Y. (2019). LSD1 inhibition promotes epithelial differentiation through derepression of fate-determining transcription factors. Cell Rep..

[146] Augert A., Eastwood E., Ibrahim A.H., Wu N., Grunblatt E., Basom R., Liggitt D., Eaton K.D., Martins R., Poirier J.T. (2019). Targeting NOTCH activation in small cell lung cancer through LSD1 inhibition. Sci. Signal..

[147] Sareddy G.R., Viswanadhapalli S., Surapaneni P., Suzuki T., Brenner A., Vadlamudi R.K. (2017). Novel KDM1A inhibitors induce differentiation and apoptosis of glioma stem cells via unfolded protein response pathway. Oncogene.

[148] Fang Y., Liao G., Yu B. (2019). LSD1/KDM1A inhibitors in clinical trials: Advances and prospects. J. Hematol. Oncol..

[149] Wang Q., Li Z., Wu Y., Huang R., Zhu Y., Zhang W., Wang Y., Cheng J. (2017). Pharmacological inhibition of Bmi1 by PTC-209 impaired tumor growth in head neck squamous cell carcinoma. Cancer Cell Int..

[150] Ginjala V., Nacerddine K., Kulkarni A., Oza J., Hill S.J., Yao M., Citterio E., van Lohuizen M., Ganesan S. (2011). BMI1 is recruited to DNA breaks and contributes to DNA damage-induced H2A ubiquitination and repair. Mol. Cell Biol..

[151] Jia L., Zhang W., Wang C.Y. (2020). BMI1 Inhibition Eliminates Residual Cancer Stem Cells after PD1 Blockade and Activates Antitumor Immunity to Prevent Metastasis and Relapse. Cell Stem Cell.

[152] Wang J., Xing Y., Wang Y., He Y., Wang L., Peng S., Yang L., Xie J., Li X., Qiu W. (2019). A novel BMI-1 inhibitor QW24 for the treatment of stem-like colorectal cancer. J. Exp. Clin. Cancer Res. CR.

[153] Kreso A., van Galen P., Pedley N.M., Lima-Fernandes E., Frelin C., Davis T., Cao L., Baiazitov R., Du W., Sydorenko N. (2014). Self-renewal as a therapeutic target in human colorectal cancer. Nat. Med..

[154] Srinivasan M., Bharali D.J., Sudha T., Khedr M., Guest I., Sell S., Glinsky G.V., Mousa S.A. (2017). Downregulation of Bmi1 in breast cancer stem cells suppresses tumor growth and proliferation. Oncotarget.

[155] Yang T., Chen Y., Zhao P., Xue H., You J., Li B., Liu Y., He C., Zhang X., Fan L. (2018). Enhancing the therapeutic effect via elimination of hepatocellular carcinoma stem cells using Bmi1 siRNA delivered by cationic cisplatin nanocapsules. Nanomed. Nanotechnol. Biol. Med..

[156] Qi S., Li B., Yang T., Liu Y., Cao S., He X., Zhang P., Li L., Xu C. (2014). Validation of Bmi1 as a therapeutic target of hepatocellular carcinoma in mice. Int. J. Mol. Sci..

[157] Kong Y., Ai C., Dong F., Xia X., Zhao X., Yang C., Kang C., Zhou Y., Zhao Q., Sun X. (2018). Targeting of BMI-1 with PTC-209 inhibits glioblastoma development. Cell Cycle.

[158] Agarwal P., Jackson S.P. (2016). G9a inhibition potentiates the anti-tumour activity of DNA double-strand break inducing agents by impairing DNA repair independent of p53 status. Cancer Lett..

[159] Seier J.A., Reinhardt J., Saraf K., Ng S.S., Layer J.P., Corvino D., Althoff K., Giordano F.A., Schramm A., Fischer M. (2021). Druggable epigenetic suppression of interferon-induced chemokine expression linked to MYCN amplification in neuroblastoma. J. Immunother. Cancer.

[160] Kelly G.M., Al-Ejeh F., McCuaig R., Casciello F., Ahmad Kamal N., Ferguson B., Pritchard A.L., Ali S., Silva I.P., Wilmott J.S. (2021). G9a inhibition enhances checkpoint inhibitor blockade response in melanoma. Clin. Cancer Res..

[161] Kato S., Weng Q.Y., Insco M.L., Chen K.Y., Muralidhar S., Pozniak J., Diaz J.M.S., Drier Y., Nguyen N., Lo J.A. (2020). Gain-of-function genetic alterations of G9a drive oncogenesis. Cancer Discov..

[162] Ishiguro K., Kitajima H., Niinuma T., Maruyama R., Nishiyama N., Ohtani H., Sudo G., Toyota M., Sasaki H., Yamamoto E. (2021). Dual EZH2 and G9a inhibition suppresses multiple myeloma cell proliferation by regulating the interferon signal and IRF4-MYC axis. Cell Death Discov..

[163] Alagoz M., Katsuki Y., Ogiwara H., Ogi T., Shibata A., Kakarougkas A., Jeggo P. (2015). SETDB1, HP1 and SUV39 promote repositioning of 53BP1 to extend resection during homologous recombination in G2 cells. Nucleic Acids Res..

[164] Fukuda K., Shinkai Y. (2020). SETDB1-mediated silencing of retroelements. Viruses.

[165] Griffin G.K., Wu J., Iracheta-Vellve A., Patti J.C., Hsu J., Davis T., Dele-Oni D., Du P.P., Halawi A.G., Ishizuka J.J. (2021). Epigenetic silencing by SETDB1 suppresses tumour intrinsic immunogenicity. Nature.

[166] Zhang S.M., Cai W.L., Liu X., Thakral D., Luo J., Chan L.H., McGeary M.K., Song E., Blenman K.R.M., Micevic G. (2021). KDM5B promotes immune evasion by recruiting SETDB1 to silence retroelements. Nature.

[167] Jayabal P., Ma X., Shiio Y. (2021). EZH2 suppresses endogenous retroviruses and an interferon response in cancers. Genes Cancer.

[168] Morel K.L., Sheahan A.V., Burkhart D.L., Baca S.C., Boufaied N., Liu Y., Qiu X., Canadas I., Roehle K., Heckler M. (2021). EZH2 inhibition activates a dsRNA-STING-interferon stress axis that potentiates response to PD-1 checkpoint blockade in prostate cancer. Nat. Cancer.

[169] Roulois D., Loo Yau H., Singhania R., Wang Y., Danesh A., Shen S.Y., Han H., Liang G., Jones P.A., Pugh T.J. (2015). DNA-demethylating agents target colorectal cancer cells by inducing viral mimicry by endogenous transcripts. Cell.

[170] Chabanon R.M., Rouanne M., Lord C.J., Soria J.C., Pasero P., Postel-Vinay S. (2021). Targeting the DNA damage response in immuno-oncology: Developments and opportunities. Nat. Rev. Cancer.

[171] Scheer S., Zaph C. (2017). The lysine methyltransferase G9a in immune cell differentiation and function. Front. Immunol..

